# A national survey of neuropsychiatry training experiences

**DOI:** 10.1192/bjb.2025.34

**Published:** 2026-04

**Authors:** Harry Costello, Matthew Baum, Cameron Watson, James B. Badenoch, Ella Burchill, Jonathan P. Rogers, Rachel Thomasson, David Okai, Timothy R. Nicholson, Anthony David, Eileen M. Joyce, Michael Dilley, Graham Blackman

**Affiliations:** 1 Division of Psychiatry, University College London, London, UK; 2 Department of Psychiatry, Brigham and Women’s Hospital, Boston, Massachusetts, USA; 3 Department of Psychiatry, Harvard Medical School, Boston, Massachusetts, USA; 4 Institute of Psychiatry, Psychology and Neuroscience, King’s College London, London, UK; 5 Department of Neurology, Manchester Centre for Clinical Neurosciences, Manchester, UK; 6 Queen Square Institute of Neurology, University College London, London, UK; 7 Department of Psychiatry, University of Oxford, Oxford, UK

**Keywords:** Neuropsychiatry, education and training, clinical neurology, survey statistics/methods, organic syndromes

## Abstract

**Aims and method:**

Neuropsychiatry training in the UK currently lacks a formal scheme or qualification, and its demand and availability have not been systematically explored. We conducted the largest UK-wide survey of psychiatry trainees to examine their experiences in neuropsychiatry training.

**Results:**

In total, 185 trainees from all UK training regions completed the survey. Although 43.6% expressed interest in a neuropsychiatry career, only 10% felt they would gain sufficient experience by the end of training. Insufficient access to clinical rotations was the most common barrier, with significantly better access in London compared with other regions. Most respondents were in favour of additional neurology training (83%) and a formal accreditation in neuropsychiatry (90%).

**Clinical implications:**

Strong trainee interest in neuropsychiatry contrasts with the limited training opportunities currently available nationally. Our survey highlights the need for increased neuropsychiatry training opportunities, development of a formalised training programme and a clinical accreditation pathway for neuropsychiatry in the UK.

The clinical discipline of neuropsychiatry is a branch of psychiatry primarily focused on disorders at the intersection of neurology and psychiatry. It emerged as a subspecialty in the latter half of the 20th century, coinciding with the divergence of training pathways in neurology and psychiatry. The increasing understanding of the comorbidity^1^ and shared biological, psychological and social underpinnings of psychiatric and neurological disorders^2,3^ has led to calls to close the divide between neurology and psychiatry and embrace greater curricular overlap during training across these specialties.^4^ This is reflected in an expanding interest and demand for integrated neuropsychiatry training among neurology and psychiatry trainees globally that is not reflected by existing training programmes in most countries.^5^

In the UK there is no dedicated training programme or formal qualification in neuropsychiatry. However, this is not the case in every country. For example, the USA requires 2 months of neurology training for all psychiatry trainees and there are a large number of accredited fellowship programmes for subspecialty certification in neuropsychiatry, and a small number of accelerated joint training programmes that enable trainees to become double board-certified in both neurology and psychiatry.^6–8^ Psychiatric training in Germany includes 1 year of neurology and vice versa.^7,9^ In contrast, the UK has an informal ‘apprenticeship model’ of neuropsychiatry training whereby trainees seek out relevant clinical experience, for example neuropsychiatry clinical and academic placements or placements in related specialist services, such as memory clinics and psychiatric liaison teams, to become appointable as neuropsychiatrists after training.^10^

The availability and demand for neuropsychiatry training in the UK and how this differs by region is currently unknown. Additionally, in the absence of a dedicated training programme, the opportunities and challenges that trainees face in accessing neuropsychiatric training remain unclear. To address these issues, we conducted the largest and most comprehensive national survey of neuropsychiatry training experiences of psychiatry trainees in the UK.

## Method

An online survey was conducted between August and December 2023 using Qualtrics (https://www.qualtrics.com). The survey was open to current psychiatry trainees in the UK and no incentive was offered for participation. The survey was disseminated to all regional training programmes in the UK via their respective training programme directors. Written informed consent was obtained from all participants. Ethical approval was obtained from King’s College London (MRA-22/23-38875). Qualifications and level of training and stage of training were recorded. Interest in a career in neuropsychiatry and access to training were assessed using a 5-point Likert scale to indicate level of agreement (‘strongly disagree’ to ‘strongly disagree’) with several statements. Survey items covered three main areas: training experience and interest in neuropsychiatry, access and barriers to training, and opportunities for change. We also acquired information on how trainees acquired neuropsychiatry and neurology experience (e.g. special interest sessions), the clinical setting (e.g. general hospital setting), their plans to arrange additional training and challenges in accessing training. We further explored interest in the development of a neuropsychiatry network and a formal qualification in neuropsychiatry. Qualitative free-text responses were collated regarding accessing neuropsychiatric training and potential changes respondents would like to see in neuropsychiatry training.

Based on an *a priori* hypothesis of geographical disparity in training opportunities, logistic regression was performed to investigate the relationship between subjective neuropsychiatry training experience in London versus all other regions. All statistical analyses were performed in R version 4.1.2. The R package ‘stats’ (version 4.1.2 for MacOS Sonoma 14.1.1, R Foundation for Statistical Computing, Vienna, Austria; see https://www.r-project.org/) was used for logistic regression modelling.

## Results

### Respondent characteristics

In total, 185 trainees took part in the study, of whom 142 (76.8%) completed at least 80% of the survey. Most responses were collected from the North West (22.6%) and London (17.4%) regions. The vast majority of respondents were higher (specialty) trainees (53.2%) or core trainees in psychiatry (45.5%), with a small number of foundation trainees (1.3%). See Table 1 for details.

**Table 1:** Survey respondents’ (*n* = 185) characteristics and description of training experience and plans in neuropsychiatry

Survey item	Frequency, *n*	Percentage, %
Training grade		
Foundation trainee	2	1.3
Core trainee	71	45.5
Higher trainee	83	53.2
Region		
East of England	5	3.2
London	27	17.4
Midlands	24	15.5
North East	10	6.5
North West	35	22.6
Northern Ireland	2	1.3
Scotland	15	9.7
South East	19	12.3
South West	17	11.0
Wales	1	0.6
Have you undertaken a formal training position in neuropsychiatry?		
Yes	61	41.2
No	87	58.8
In what role did you gain training in neuropsychiatry?		
Clinical training post	21	30
Special interest session	18	25.7
Out-of-training experience	12	17.1
Other	19	27.1
What do you feel are the general challenges to accessing training opportunities in neuropsychiatry?		
Insufficient rotations	130	42.1
Lack of advertising	51	16.5
Challenges travelling to placements	28	9.1
Lack of encouragement	49	15.9
Lack of confidence	33	10.7
Other	18	5.8
Do you plan to gain further neuropsychiatry training?		
Yes	40	33.1
No	81	66.9
Have you had experience working in neurology?		
Yes	33	23.2
No	109	76.8
Type of experience working in neurology		
Foundation training	20	50
Core medical training	8	20
Trust grade/clinical fellow	5	12.5
Higher trainee	1	2.5
Other	6	15
Do you think there should be a formal accreditation in neuropsychiatry?		
Yes	125	90.6
No	13	9.4

### Training experience and interest in neuropsychiatry

Free-text survey responses from trainees on training experience and interest in neuropsychiatry included:

‘We have had dedicated teaching from neuropsychiatrists and a neurologist as part of the regional teaching, which was really valuable. It would be great to have opportunities for clinical experience.’ (Core trainee, North West region)

‘I am very interested in training in neuropsychiatry, but the lack of guidance and opportunities is really frustrating.’ (Core trainee, North West region)

Most respondents had not undertaken a formal training post in neuropsychiatry (58.8%). Of those who had undertaken such training, most gained neuropsychiatry experience through clinical training posts (30%) or special interest sessions (25.7%). One-third of respondents planned to gain further neuropsychiatry training, and just under half (43.6%) were interested in following a career in neuropsychiatry (Table 1, Fig. 2).

**Fig. 1:**
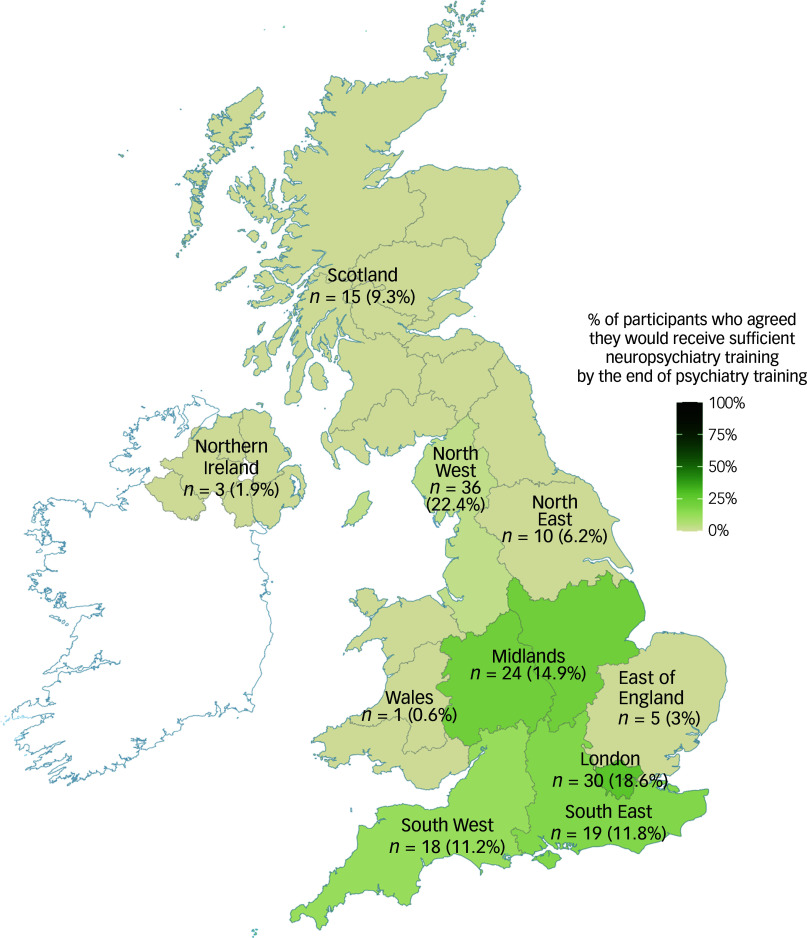
Survey responses from across the UK (*n*, number of responses from each region; %, percentage of total responses) and proportion of participants from each region who agreed that they would receive sufficient neuropsychiatry experience by the end of their training.

**Fig. 2:**
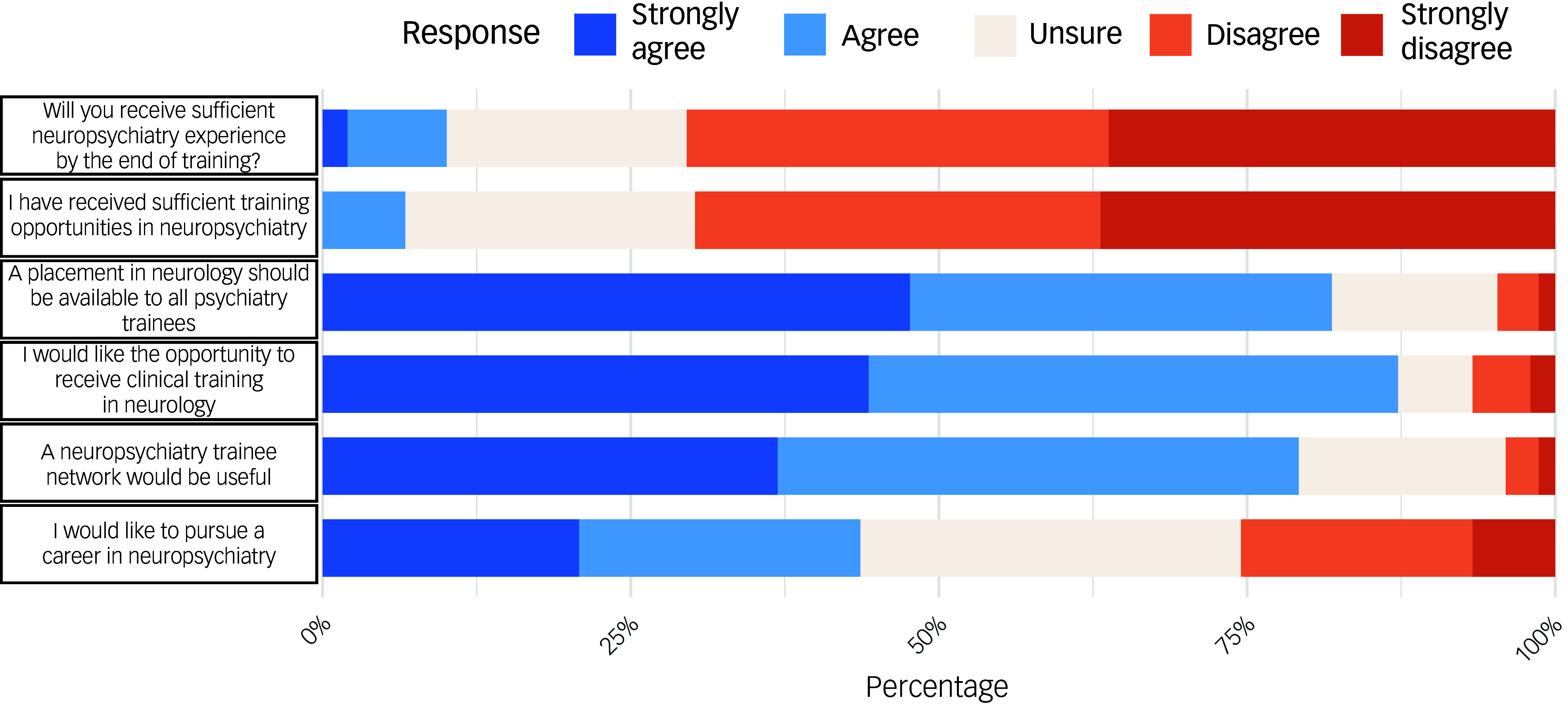
Responses regarding neuropsychiatry training experience, opportunities and future plans. Responses were made on a 5-point Likert scale, ranging from ‘strongly disagree’ to ‘strongly agree’.

A quarter of respondents had experience of working in neurology, most commonly during foundation training (50%). The vast majority of participants agreed or strongly agreed that a clinical placement in neurology should be available to all psychiatry trainees (81.9%).

### Access and barriers to training in neuropsychiatry

Free-text survey responses from trainees on access and barriers to training in neuropsychiatry included:

‘There has been no opportunity to explore or gain exposure to neuropsychiatry during my training to date – I think this results in a lack of skills and confidence in this area. I don’t think there are any training posts within my deanery.’ (Higher trainee, South East region)

‘Lack of exposure during training, therefore limited interest.’ (Higher trainee, North West region)

Only 10% of respondents agreed or strongly agreed that they would receive sufficient neuropsychiatry training by the end of training (Fig. 2). Trainees in London (18.6%) and the North West (22.6%) were more likely to report that they felt that they would receive sufficient neuropsychiatry training (Fig. 1), but even within these regions less than a quarter of respondents agreed. There was a significant association between training in London versus all other regions, with respondents agreeing they would receive sufficient neuropsychiatry training by the end of psychiatry training (odds ratio OR = 6.25, 95% CI 1.45–27.8, *P* = 0.013).

Insufficient availability of training rotations, inadequate advertisement or awareness of opportunities, and a lack of encouragement by senior clinicians were identified as the most common challenges to accessing neuropsychiatry training. This was supported by qualitative responses in which a recurrent theme was that the regionally dependent access to training rotations limited the development of clinical interest and confidence in neuropsychiatry.

### Opportunities for change

Free-text survey responses from trainees on opportunities for change in UK neuropsychiatry training included:

‘I would like to see clearer guidance on how to develop a career in this area, particularly in regions where there are not currently neuropsychiatrists.’ (Higher trainee, North East region)

‘A formal accreditation would make training more tangible and clarify what is expected/required.’ (Core trainee, North East region)

‘Formal accreditation would only be helpful if there are enough training opportunities available for trainees to have equal access.’ (Higher trainee, North West region)

‘More high quality teaching resources for neuropsychiatry are needed, it is an area which lots of trainees struggle with during exams.’ (Core trainee, South East region)

‘More collaboration between neurology and psychiatry trainees, more easily accessible accreditation as part of formal training pathway, and better distribution across the UK or access that can involve a blend of remote and in person training to allow for involvement of other deaneries that don’t have tertiary centres.’ (Core trainee, South West region)

Over 90% of respondents agreed that a formal accreditation in neuropsychiatry should be offered. Respondents reported that an accreditation process would provide further structure, guidance and clarity as to the skills and training required to specialise in neuropsychiatry. However, a caveat identified by some respondents was the need for regional equity in access to neuropsychiatry training posts and other training opportunities that are required for accreditation. Survey respondents also encouraged inter-deanery collaboration and remote access opportunities for trainees to access neuropsychiatry training in regions with more established and wider neuropsychiatric service provision to enable more equitable access nationally.

Three-quarters of respondents supported the creation of a neuropsychiatry trainee network. Those who supported a network reported that the main benefits of this would be to meet other neuropsychiatrists and trainees interested in neuropsychiatry and improve access to resources, training opportunities and career guidance.

Other themes identified in free-text responses included an interest in early collaboration between neurology and psychiatry trainees, and increased flexibility, with training schemes to be supported to apply for out-of-programme training posts in neurology.

## Discussion

We conducted the largest and most comprehensive survey of neuropsychiatry training experiences in the UK to date. Our findings highlight the strong demand for neuropsychiatry training among psychiatry trainees, the widespread barriers many trainees face, and the opportunities for improvement in neuropsychiatry training across the UK.

### Barriers to neuropsychiatry training

Despite most respondents expressing an interest in gaining training and clinical experience in neuropsychiatry, only one in ten reported that they felt they would receive sufficient training opportunities by the time they became a consultant. The most frequently perceived barriers to accessing neuropsychiatry training were insufficient availability of clinical placements, a lack of exposure to neuropsychiatry during training, inadequate awareness of training opportunities and insufficient encouragement by senior clinicians. These barriers align with previous survey results reported by trainees in other countries, indicating that deficiencies in neuropsychiatry training are not unique to the UK.^5^ However, in other European countries, such as Germany, Belgium and Austria, neuropsychiatry is an integrated and mandatory component of training for neurologists and psychiatrists.^10^ These alternative models of training emphasise the integration of neurology and psychiatry, which is reflected in the perceived access to training resources reported by trainees.

Our survey findings also highlight regional variation and inequitable access to neuropsychiatry training across the UK. This disparity is most apparent in comparison with London, where trainees have greater exposure and access to neuropsychiatry clinical posts. This may reflect neuropsychiatric services generally having developed in large clinical-academic neuroscience centres developed in part to receive national referrals, which have been historically concentrated in London. Neuropsychiatry services outside London are rarely assigned dedicated training posts, including those housed within large regional neurosciences centres. Given that most clinicians do not move from the geographical region they train in for their consultant post,^11^ the regional disparity in neuropsychiatry training is likely to be a barrier to the development of neuropsychiatric services outside of these centres.

### Suggestions for change

To address barriers and regional variation in access to training, several initiatives were clearly supported by survey respondents. A formalised accreditation process with an associated curriculum and expectations of clinical experience during training would provide clarity on the skills and expertise that are expected to pursue a career in neuropsychiatry. Accreditation may also encourage regional deaneries and national training policymakers to improve access to training rotations or support out-of-programme clinical fellowships.

The majority of respondents also supported the development of a trainee network offering access to neuropsychiatry teaching, mentorship and support for trainees to gain clinical experience of neuropsychiatry that is not confined to specific training regions.

Finally, the adoption of models used in countries where neurology and psychiatry training are more integrated was supported by over three-quarters of trainees. An amount of neurology training for psychiatrists is mandated in some other countries, for example notably Germany and the USA. In contrast, access to neurology training for psychiatry trainees in the UK is currently limited, owing to the segregated and siloed structure of specialty training. However, pilot schemes of collaborative training programmes between old age psychiatry and geriatric medicine in the UK have shown a positive effect on clinical confidence and expertise for both trainee groups and demonstrate a feasible pathway of improving access to cross-specialty training within existing training structures.^10^ There are also examples of third-sector organisations funding clinical fellowship opportunities where psychiatry trainees work in neurology teams. For example, the charity Parkinson’s UK, recognising the need for psychiatrists with expertise in movement disorders, has pioneered the funding of advanced clinical fellowships where trainees are supported to work in and develop expertise in movement disorder services. This model of formal, advanced clinical fellowships after completion of general psychiatry training has also been successfully applied in the USA, with over 44 accredited programmes. However, these kinds of opportunity in the UK currently are limited and require the support of training institutions and flexibility of training programmes to accommodate trainees gaining neuropsychiatry experience outside of formal training rotations.

It has been nearly 40 years since the definition of neuropsychiatry was debated at the inaugural meeting of the British Neuropsychiatry Association, held in conjunction with its sister organisation, the American Neuropsychiatric Association.^12^ Since this meeting, there have been repeated calls for the integration and expansion of neuropsychiatry training in the UK. However, while other subspecialties, such as liaison psychiatry, have established clear career pathways with widely available specialist training programmes, neuropsychiatry often remains viewed as an esoteric career restricted to tertiary clinical academic centres. The prevalence and neuropsychiatric needs of conditions such as functional neurological disorder,^13^ traumatic brain injury,^14^ encephalitis,^15^ Parkinson’s disease^16^ and epilepsy^17^ are expanding, and more neuropsychiatrists are needed to meet this demand. Improved neuropsychiatry training partly relies on making the case for and establishing neuropsychiatric services more widely. A clear definition of neuropsychiatry that delineates what the specialty offers that others cannot, and its growing clinical need, is crucial in communicating to commissioning bodies and institutions that neuropsychiatry training is an essential component of psychiatry’s future.

In summary, our findings suggest a strong interest in neuropsychiatry among trainees in the UK. However, current training opportunities do not meet the demand. There was significant geographical variability in the availability of these opportunities. The expansion of clinical exposure to neuropsychiatry, alongside the development of a formalised training programme and a clinical qualification in neuropsychiatry, would be welcomed to meet the growing demand while ensuring the highest clinical standards are maintained.

### Strengths and limitations

To our knowledge, this is the largest and most comprehensive survey of neuropsychiatry training experiences conducted in the UK. To ensure a broad range of perspectives, the survey was disseminated through all the deaneries. The anonymity provided encouraged open and candid responses. By including several questions requiring free-text responses, we were able to deepen the scope of our exploration.

A limitation of this study is the potential for response bias, as not all trainees may have had equal access to the survey. Additionally, the method of dissemination prevented us from determining the total number of trainees to whom the survey was distributed. However, this risk was partially mitigated by distributing the survey to all training programmes across the UK. Additionally, a degree of self-selection is likely to have occurred, as doctors with a particular interest in neuropsychiatry may have been more inclined to participate. Survey fatigue also posed a challenge, leading to incomplete responses. We attempted to mitigate this by keeping the survey brief; as a result, the majority of respondents completed at least 80% of the survey.

## Data Availability

The data that support the findings of this study are available from the corresponding author, H.C., on reasonable request.

## About the authors

**Harry Costello** is an Old Age Psychiatry registrar and National Institute for Health and Care Research (NIHR) clinical lecturer at the University College London (UCL) Division of Psychiatry, London, UK. **Matthew Baum** is a neuropsychiatrist at Brigham and Women’s Hospital, Boston, Massachusetts, USA, and Harvard Medical School, Boston, Massachusetts, USA, and director of the Translational Neuroimmune Psychiatry Research Program in the Brigham and Women’s Hospital’s Department of Psychiatry. **Cameron Watson** is an NIHR academic clinical fellow and core psychiatry trainee at the Institute of Psychiatry, Psychology & Neuroscience, King’s College London, London, UK. **James B. Badenoch** is an NIHR academic clinical fellow and core psychiatry trainee at the Institute of Psychiatry, Psychology & Neuroscience, KCL, UK. **Ella Burchill** is an NIHR academic clinical fellow and core psychiatry trainee at the UCL Division of Psychiatry, London, UK. **Jonathan P. Rogers** is a general adult psychiatry registrar and NIHR clinical lecturer at the UCL Division of Psychiatry, London, UK. **Rachel Thomasson** is a consultant neuropsychiatrist at Salford Royal Hospital, Salford, UK. **David Okai** is a consultant neuropsychiatrist at South London and Maudsley NHS Foundation Trust, London, UK. **Timothy R. Nicholson** is a consultant neuropsychiatrist and clinical senior lecturer at the Institute of Psychiatry, Psychology & Neuroscience, King’s College London, London, UK. **Anthony David** is a consultant neuropsychiatrist, professor of mental health and director of the UCL Institute of Mental Health, London, UK. **Eileen M. Joyce** is a consultant neuropsychiatrist and professor of neuropsychiatry at the UCL Institute of Neurology, London, UK. **Michael Dilley** is a consultant neuropsychiatrist at King’s College Hospital, London, UK. **Graham Blackman** is an NIHR academic clinical lecturer at the Department of Psychiatry, University of Oxford, Oxford, UK.
